# Solitary Myocardial Metastasis from Locoregionally Controlled Squamous Cell Carcinoma of the Oral Cavity

**DOI:** 10.7759/cureus.650

**Published:** 2016-06-21

**Authors:** Kevin Martell, Roderick Simpson, David Skarsgard

**Affiliations:** 1 Oncology, University of Calgary; 2 Department of Pathology and Laboratory Medicine, University of Calgary

**Keywords:** distant metastasis, heart, head and neck cancers, scc (squamous cell cancer), oligometastatic

## Abstract

We present the case of a 62-year-old male originally diagnosed with squamous cell carcinoma (SCC) of the right retromolar trigone, Stage cT2N2bM0. He was treated radically with a pharyngotomy and segmental mandibular resection, right selective neck nodal dissection, and then reconstruction with a free fibular flap. The pathologic stage was T4aN1. He then received adjuvant chemoradiation therapy with a radiation dose of 6,000 cGy in 30 fractions, along with cisplatin, 100 mg/m^2^ every three weeks.

Good local control was repeatedly documented for two years. He then presented with shortness of breath and new-onset atrial fibrillation (AF) with rapid ventricular response. Computed tomography/pulmonary embolus protocol (CT/PE) showed no evidence of pulmonary embolism but did show a small pericardial effusion. His AF was refractory to medical management, and he was later admitted to hospital with congestive heart failure. He was found to have a large mass arising from the free wall of the right ventricle, a biopsy of which confirmed squamous cell carcinoma consistent with his head and neck primary. The patient declined further therapy and passed away within one month of presentation.

This case is unusual in that the only known site of metastatic disease seen was to the myocardium of the right ventricle, presenting as cardiac arrhythmia and congestive heart failure. Although post-mortem studies show cardiac metastases to occur in 2 to 20% of cancer patients, it is rarely seen as a sole site of relapse in clinical practice.

## Introduction

In most malignancies, cardiac metastases occur at rates between 2 and 20% [1-2]. For head and neck cancers, the incidence appears to be lower (_~_ 1%) [3]. In all cases, there are four basic processes through which tumors can spread to the heart: direct extension, bloodstream drop metastasis, lymphatic spread to the heart, and intracavitary diffusion through the inferior vena cava or pulmonary veins [1, 4]. These are usually clinically silent tumors which present in patients with uncontrolled systemic disease. In fact, most cardiac metastases remain clinically silent until the death of the patient from systemic disease elsewhere [4].

Hence, the presentation of a head and neck cancer patient with an isolated metastasis to the myocardium is a rare occurrence with little description in the literature. We present one such case that we recently encountered at our centre, in which the patient's disease had been controlled locoregionally for two years prior to his presentation with a cardiac metastasis causing heart failure.

Written and informed consent were obtained from the patient’s next of kin prior to the presentation of this case report. Informed patient consent was obtained at the time of treatment.

## Case presentation

A 60-year-old non-smoking white male, with an alcohol history of 2-3 drinks per day, was diagnosed in July 2013 with squamous cell carcinoma (SCC) of the right retromolar trigone with extension into the adjacent buccal mucosa. He was treated with primary surgery on September 12, 2013, consisting of a right lateral pharyngotomy and segmental resection of the right posterior mandible and adjacent buccal mucosa, and a right selective neck dissection followed by left free fibula osteoseptocutaneous flap reconstruction. Final pathology showed metastatic keratinizing SCC measuring 0.96 cm with a 0.5 cm depth of invasion. There was an invasion of the bony mandible into the cortex and medulla. There was no lymphovascular space or perineural invasion and all margins were free of cancer, with the closest being 0.18 cm medially. One of 12 lymph nodes in right Level I and one of one facial lymph nodes were positive for metastatic disease, the latter with extranodal extension. No cancer was found in any of 20 lymph nodes from Levels 2-4. Pathologic stage was T4aN2b with an R0 resection.

After multidisciplinary tumor board discussion, it was recommended that he receive adjuvant radiation therapy (RT) with concurrent cisplatin chemotherapy. Starting six weeks postoperatively, he received a radiation dose of 6,000 cGy in 30 fractions over six weeks to the tumor bed and involved nodal regions via a 5-field IMRT plan using 6 MV photons, along with a q3 weekly bolus of cisplatin, 100mg/m^2^.

He completed all adjuvant treatment on December 3, 2013, uneventfully and without unplanned delays or unexpected toxicities. His last clinical follow-up appointment on January 28, 2015 revealed no evidence of locoregional recurrence and no significant late treatment toxicities.

In February 2015, he presented to the emergency room with acute shortness of breath, palpitations, and a recent history of air travel. Electrocardiogram (ECG) showed rapid atrial fibrillation. CT scan of the chest, using a pulmonary edema (PE) protocol, showed a small pericardial effusion, measuring up to 1.3 cm in width, and moderate-sized, uncomplicated bilateral pleural effusions. There was a single 0.9 cm short axis prevascular lymph node, which was felt most likely to be reactive. He was discharged with arrangements for an outpatient cardiology follow-up. His atrial fibrillation proved to be difficult to control, in part because of intolerance of beta blockers due to pre-existing asthma. On July 1, 2015, he re-presented with shortness of breath on exertion and peripheral edema. Initial bloodwork revealed the N-terminal prohormone of brain natriuretic peptide (NT-proBNP) was elevated at 1650 (0-300) with mild high sensitivity troponin elevation at 20 (0-14). ECG showed a septal infarct, right axis deviation, and a marked ST segment abnormality. The rhythm was variant between sinus tachycardia and atrial flutter. After admission, an echocardiogram was performed, and a large mass was identified in the right ventricle.

**Figure 1:**
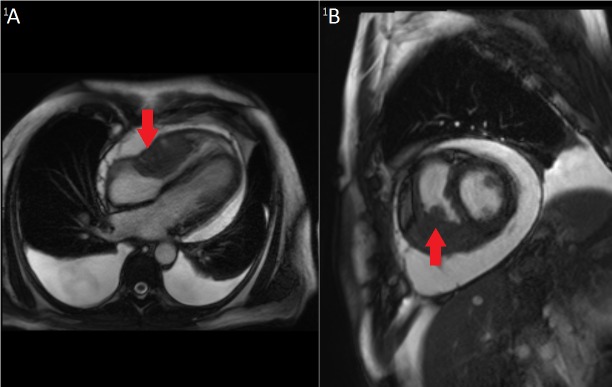
Cardiac MRI of the Tumor A: Axial T2-weighted and B: Sagittal T2-weighted MRI images showing large tumor (arrows) in the right ventricle obstructing flow through the tricuspid valve and extending into the right atrium. Additional findings included a pericardial effusion and bilateral pleural effusions.

The following day he underwent pulmonary function tests (PFTs), which showed both FVC and FEV1 < 50% of predicted values. A CT scan of the neck showed stable post-treatment changes and no evidence of primary or residual tumor, while CT of the chest, abdomen, and pelvis revealed the growth of the prevascular mediastinal lymph node to 1.7 x 1.2 cm. Also noted were hepatic venous congestion and the right ventricular mass. Retrospectively, this was thought to have been visible as a small abnormality on the CT/PE study from 4.5 months earlier. Cardiac MR (Figures 1A-1B) showed the mass to be 6.7 x 3.8 x 4.5 cm and centered on the right ventricular free wall. There was a narrowing of the tricuspid valve and mass extension into the right atrium. A large pericardial effusion was associated with this mass.

A biopsy was attempted via right heart catheterization through the right internal jugular vein under transthoracic echocardiogram guidance. Repeated attempts were unsuccessful and yielded only normal myocardial tissue because of catheter deflection as it entered the right ventricle. Diagnostic thoracentesis of his left pleural effusion showed only rare atypical cells.

**Figure 2:**
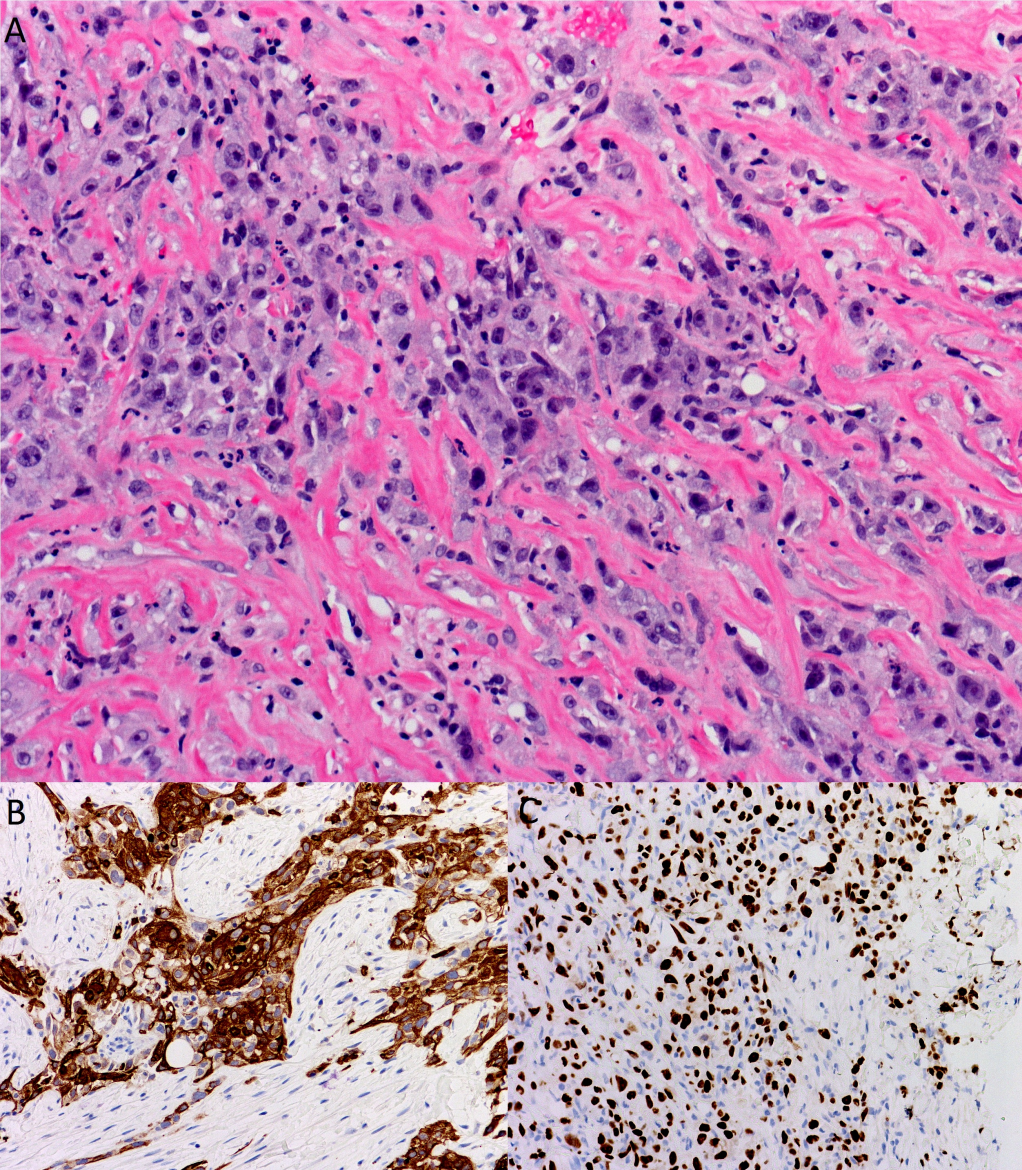
Myocardial Metastasis Histology Representative slices of A: H&E stain showing squamous cell carcinoma embedded in the myocardial wall; B: CK 5/6 staining (strongly positive); and C: p63 staining of myocardial metastasis (positive).

After much consideration and discussion with the patient about potential benefits and risks, it was decided to proceed with an open biopsy. On July 14, 2015, he underwent sternotomy with direct visualization and biopsy of the pericardium and the right ventricular mass. Pathology showed metastatic poorly differentiated squamous cell carcinoma arising from within the myocardium of the right ventricular free wall (Figures 2A-2C). A full immunohistochemical panel revealed the tumor to be p63, 34BE12, E-cadherin, pancytokeratin (PCK), and cytokeratin 5/6 (CK5/6)-positive. It was negative for melanin, S100, WT1, CD34, and CD45 and, hence, felt to be consistent with metastatic disease from his original oral cancer.

Shortly after biopsy, palliative radiotherapy with 2,000 cGy in five fractions was discussed with the patient. The goals of treatment would have been to try and improve his cardiac function. Risks were discussed, including potential worsening of arrhythmias and catastrophic cardiac rupture due to regression of a tumor that was replacing much of the cardiac wall. He appreciated the risks and uncertain benefits and consented to treatment as described. Unfortunately, after discharge, he began decompensating again and, hence, pursued comfort measures only. He passed away within one week of this decision and exactly one month after presenting in heart failure.

## Discussion

In a review of the literature, six studies of patients presenting with symptomatic cardiac metastases were found. Primary sites of malignancy and primary pathologies varied, but in all cases, the patients died [2, 5–9]. The case we present represents the furthest out from primary treatment in which such an occurrence has been documented, the largest reported such tumor, and one of four reports where other systemic disease is minimal.

Rivkin, et al. presented the case of a 57-year-old male with a 1.2 cm lesion in the right ventricle from a right base of tongue primary [5]. Unfortunately, this patient had extensive disease on subsequent CT scan and was not a resection candidate. He proceeded on to chemotherapy but died several months later from systemic disease.

Other reported cases include two cases from Nagata, et al. of metastases to the pericardium, which proved fatal [7], and a report from Werbel, et al. of a 61-year-old white woman with a prominence in the right ventricle pathologically shown to be metastatic from a base of the tongue primary [2]. In another report, Cheruvu, et al. described a man with metastatic adenocarcinoma of an unconfirmed primary [8]. Ejection fraction was preserved, but the patient eventually died, presumably of systemic disease.

Furthermore, Schwender, et al. reported on a 73-year-old female with a buccal mucosal primary [6]. Like our case, this patient had several episodes of rapid atrial fibrillation but began six months after the completion of chemoradiotherapy to the primary. Echocardiogram showed an adherent lesion to the pericardium, and chest radiograph showed an additional 2 cm lingular lung nodule. The patient died, and at autopsy, they found additional lung, thyroid, and lymphatic metastases.

Resection of cardiac metastases has been described, but the experience is limited and it is unclear whether this is a life-prolonging measure [7]. The role of RT is arguably even less clear. In a recently published review, Ghiam, et al. reported response in five out of 10 patients receiving palliative RT for cardiac metastases [9]. However, none of these tumors was from head and neck primaries and no patient had squamous cell carcinoma. Furthermore, only two patients had cardiac metastases as an isolated site of disease. We offered our patient short course of palliative radiotherapy, but this was abandoned before it started as his condition rapidly deteriorated. This patient passed away soon after his presentation and, in hindsight, it would have been unlikely that he would have tolerated or benefitted from palliative radiotherapy, had it been given.

Reports, such as this one, are likely to be of increasing importance in the future as patient survivorship increases from advances in cancer management. With improved local control in head and neck malignancies, the prevalence of symptomatic myocardial metastases from them is likely to increase. Understanding the presentation, diagnostic challenges, and natural course of myocardial metastasis will better inform future management decisions. 

## Conclusions

We present a rare case of cardiac metastasis from a locoregionally-controlled squamous cell carcinoma from a head and neck primary. The only other evidence of systemic disease was a single prevascular mediastinal lymph node, which grew minimally over a six month time period. The patient presented initially with new onset atrial fibrillation that was difficult to control and then in fulminant congestive heart failure six months later.
